# Analysis of neurosurgical procedures with unplanned reoperation for quality improvement: A 5-year single hospital study

**DOI:** 10.1097/MD.0000000000028403

**Published:** 2021-12-30

**Authors:** Wei-Chao Huang, Yin-Ju Chen, Martin Hsiu-Chu Lin, Ming-Hsueh Lee

**Affiliations:** a Department of Neurosurgery, Chang Gung Memorial Hospital, Chia-Yi Branch, Pu Tz City, Chia-Yi, Taiwan; b College of Medicine, Chang Gung University, Tao-Yuan, Taiwan.

**Keywords:** neurosurgery, quality indicator, unplanned reoperation

## Abstract

The unplanned return to the operating room rate is a quality metric for assessing hospital performance. This study aimed to evaluate the cause, incidence, and time interval of unplanned returns in index neurosurgical procedures within 30 days of the initial surgery as an internal audit. We retrospectively analyzed neurosurgical procedures between January 2015, and December 2019, in a single regional hospital. The definition of an unplanned return to the operating room was a patient who underwent two operations within 30 days when the second procedure was not planned, staged, or related to the natural course of the disease.

A total of 4365 patients were identified in our analysis, of which 93 (2%) had an unplanned return to the operating room within 30 days of their initial surgery during admission. The most common reason for an unplanned return to the operating room for a cranial procedure was hemorrhage, followed by hydrocephalus and subdural effusion, which accounted for 49.5%(46/93), 12%(11/93), and 5.4%(5/93) of cases, respectively. In spinal procedures, the most common cause of return was a residual disc, followed by surgical site infection, which accounted for 5.4%(5/93) and 4.3%(4/93) of cases, respectively. The overall median time interval for unplanned returns to the operating room was 3 days (interquartile range, 1–9).

Lowering the rate of postoperative hemorrhage in cranial surgery and postoperative residual disc in spine surgery was crucial as an internal audit in a 5-year single institute follow-up. However, the unplanned reoperation rate is less helpful in benchmarking because of the heterogeneity of patients between hospitals.

## 1 Introduction

In recent years, technological advancements in health care and the emphasis placed on risk management and surgical quality have reduced avoidable patient suffering and biopsychosocial impacts.[^1^,^2^] A morbidity and mortality review of the rate of unplanned return to the operating room is crucial for improving healthcare quality. It is a valuable tool for assessing surgical adverse events and is a helpful metric for determining the quality of surgical care in many surgical subspecialties.[^3^,^4^,^5^] The incidence rate of adverse events for admitted patients is 5.7% to 9%, and surgical adverse events account for 39.6% to 51.4% of the overall in-hospital adverse event rate.[^6^,^7^,^8^,^9^,^10^]

Few studies have evaluated the rate of unplanned return to the operating room in the neurosurgical department.[^11^,^12^,^13^,^14^,^15^] In addition, neurosurgical approaches and patient characteristics between different medical centers and local hospitals are diverse. Therefore, detailed analysis at each institution is required for future benchmarking.

This study aimed to use 5-year data for an internal audit to assess the reoperation rate, reoperation time interval, and preventable causes of index operations for unplanned returns to the operating room in 30 days in a single hospital.

## 2 Materials and methods

### 2.1 Patient population

In patients, who underwent neurosurgical procedures at the Chang Gung Memorial Hospital, Chiayi Branch between 2015 and 2019 were evaluated. Patients who returned to the operating room within 30 days after primary surgery were identified. Data were collected from the weekly rounds meeting for new surgical cases, and the chief resident compiled all case numbers, surgery categories, indications for the operations, and surgical methods for the week in Excel files. We defined unplanned returns to the operating room (UROR) as when the patient underwent two operations when the second procedure was not scheduled or planned before the first primary surgery and was not related to the disease's natural course. UROR surgeries did not include local anesthesia procedures (pain control, nerve block/ganglion block) or radiotherapy.

### 2.2 Data collection

We did not use a computer coding system to capture the data falling in the Clavien-Dindo classification grade 3 to avoid missing calculations or recording errors, which could have affected the validity and reliability of the data.^[16]^ We collected the patient list of surgical rounds from our department every week to ensure reliable data. We did not use the electrical medical record system software to sort our UROR cases automatically. We found that much of the documentation was incomplete, and automatic software sorting would underestimate.^[16]^ Our manual collection from the chart rounds was more reliable and was conducted by two surgeons in training and two certified neurosurgeons. We searched our electronic medical records system to obtain more detailed information.

### 2.3 Definition of planned and unplanned operations

An operation was considered planned if it was scheduled before the first primary operation. For example, when operating to treat seizures, we first implanted the electrode for detection and then arranged a second operation to remove the abnormal focus.

An unplanned operation was defined as a repeat operation that was not initially scheduled. Unplanned operations can be divided into two categories based on whether the event is related to the original surgery. Operations were considered unrelated to the original surgery when the indication for the second surgery was affected by the disease's natural course. Sometimes, the natural course of the disease worsens the condition, necessitating a second operation. For example, following emergent craniectomy operations for acute subdural hemorrhage, a delayed traumatic intracranial hemorrhage may occur after the operation. A second operation would be expected owing to the traumatic mechanism, but this operation would not have been scheduled in advance. Surgeries related to the initial operation indicated that an adverse event caused the patient's return to surgery due to surgeon-related factors, such as residual hematoma, wound infection, and instrument dislocation. In our study, we excluded those operations related to the disease's natural course because the preventable causes from surgeon-related factors are worthy of analysis.

### 2.4 Statistical analysis

The primary outcome was the rate of unplanned reoperations for each index surgery. Patient characteristics were described as the mean ± standard deviation or median with the minimum, maximum, or 25th and 75th interquartile range (IQR) for continuous variables. Categorical variables are presented as proportions (percentages). Therefore, the chi-squared test was performed for each category variable (such as surgical diagnosis and an indication of reoperations) between non-reoperation and reoperation cases. Baseline characteristics (sex, age, time interval between primary operation and complication-related reoperation) were also analyzed using the chi-squared test and Wilcoxon rank-sum test. For time-event analyses, a Cox regression analysis was performed. Statistical significance was set at *P* < .05 were considered statistically significant (2-tailed). All statistical analyses were performed using SPSS software (SPSS Statistics, version 20, IBM Corporation, USA).

## 3 Results

### 3.1 Patient demographics

A total of 4365 patients were evaluated during the five years from 2015 to 2019; 318 patients required repeat operations. Of these, 169 cases were planned returns to the operating room, 56 cases were disease nature course-related reoperations, and 93 cases were surgical site-related reoperations. The age ranged from 18 to 93 years, with a median of 59 years, and the male (2324) to female (2041) ratio was 1.13. Traumatic brain injury (n = 510) was the most common cranial surgical diagnosis, followed by brain tumor resection (n = 440), VP shunt (193), vascular surgery (n = 147), spontaneous intracranial hemorrhage removal (n = 124), and cranioplasty (n = 120). In addition, the overall spinal surgical procedures accounted for 2831 case.

Amount of 93 surgically site-related UROR cases. Age ranged from 18 to 87 years, with a median age of 64 years, and the male (78) to female (15) ratio was 5.2. The proportion of male patients increased significantly in the UROR group (*P* < .01). Brain tumors (21) and spine instrument/decompression procedures (21) were the most common surgical site related reoperations, followed by traumatic brain injury (16), spontaneous ICH (16), vascular (10), cranioplasty (4), and VP shunt (5) (Table 1).

**Table 1:** Patient demographics.

		Overall (N)	Percent	reoperation(N)	Percent	
		4365	100	89	2	
Sex	Female	2041	46.8	14	15.7	
	Male	2324	53.2	75	84.3	*P* < .01
Age	Median	59		64		
	Minimum	18		18		
	Maximum	93		87		
Length of stay						
	Median	10		25		*P* < .05
diagnosis						*P* < .01
	Trauma	510	12	16	18	
	Vascular	147	3	10	11	
	Brain tumor	440	10	21	23.5	
	Spine	2831	65	21	23.5	
	sICH	124	3	16	18	
	Cranioplasty	120	3	4	5	
	VP shunt	193	4	1	1	

The incidence rates of UROR for each surgical diagnosis were as follows: 12.9% (16/124) for spontaneous intracranial hemorrhage evacuation, 6.8% (10/147) for vascular surgery, 4.8% (21/440) for a brain tumor, 3.3% (4/120) for cranioplasty, 3.1% (16/510) for traumatic brain injury, 2.6% (5/193) for VP shunt surgery, 0.7% (21/2831) for spinal surgery. All the above-classified diagnosis were statistically significant between non-UROR patients and UROR patients (*P* < .01).

### 3.2 Causes of return to the operating room

#### 3.2.1 Cranial procedures

Hemorrhage (46) and hydrocephalus (12) are the common causes of UROR in cranial procedures. Bleeding was the leading cause of reoperation in the VP shunt (5/5, 100%), spontaneous ICH (13/16, 87%), traumatic brain injury (14/16, 82%), and vascular surgery (6/10, 60%) (Table 2). The relationship between hemorrhage and three surgical diagnoses of UROR, including traumatic brain injury, spontaneous ICH, and VP shunt, was statistically significant (*P* = .023). The association between hydrocephalus and the brain tumors with UROR was also statistically significant (*P* = .002).

**Table 2:** Causes of reoperation for cranial surgery.

	Traumatic brain injury	Vascular	Brain tumor	sICH	Cranioplasty	Vp shunt
Hemorrhage	14	6	6	13	2	5
hydrocephalus	1		8	2		
Subdural effusion	1	3	1			
Brain edema		1	1			
Others^∗^			5	1	2	

Among the 46 patients with postoperative bleeding, the VP shunt (5) insertion case resulted from hematoma over the insertion tract, spontaneous intracranial hemorrhage (13) resulting from residual hematoma and inadequate hemostasis, traumatic brain injury resulting from post-craniectomy epidural hembleeding (1), remote hemorrhage over the cerebellar (1), acute subdural hemorrhage after burr hole drainage for chronic subdural hemorrhage (2), and brain tumor cases (6) all resulted from postoperative parenchymal hemorrhage, vascular surgery resulting from hematoma around the clipping site (4), hematoma after AVM removal (1), and neck hematoma after endarterectomy (1). Cranioplasty cases result from epidural hemorrhage (1) and intraventricular hemorrhage (1).

Among the 11 patients with postoperative hydrocephalus, 8 had brain tumors, which accounted for 38% (8/21) of all brain tumor surgeries with UROR. Three were intraventricular lesions, and one was clivus chordoma from a transnasal endoscopic approach, one was a cerebellopontine angle tumor, and the other three were gliomas. In addition, one patient had bilateral subdural effusion removal after trauma and accounted for 6.25% (1/16) of traumatic brain injury with UROR. Two were the result of post-sICH removal and accounted for 12.5% (2/16) of these surgeries with UROR.

#### 3.2.2 Spinal procedures

Surgical site infection (n = 4) and residual disc (n = 5) were the main causes of UROR in spinal procedures (Table 3).

**Table 3:** Causes of reoperation for spinal surgery.

Spine tumor	Residual tumor	Graft dislodge			
	2	1			
Spine spondylosis	Residual disc	Wound infection	Hemorrhage	Inadequate decomp.	Graft dislod.
	5	4	2	3	2
Spinal abscess	Graft dislod.				
	1				
Spine trauma	Wound poor healing				
	1				

Among the 5 cases of postoperative residual disc, all were L5S1 residual discs. Among these four postoperative surgical site infection cases, all were under spinal decompression procedures. The other causes of UROR were graft dislodging in the spinal cord procedure (anterior cervical decompression and fusion), inadequate decompression (c spine laminectomy), and residual tumor (T spine epidural metastatic lesion). They have poor healing (spine trauma).

### 3.3 Timing of the return to the operating room

We analyzed the causes of reoperation following brain surgery and the time interval between the first operation and return to the operating room. We defined early return as <7 days postoperatively and late reoperation as postoperative 7 days later.

**Figure 1:**
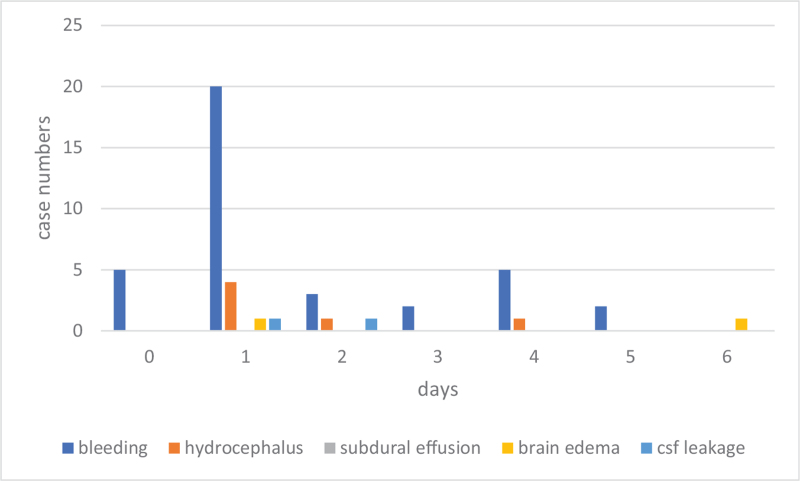
The distribution of the causes of return (cranial surgery) in early reoperation period (<7 days).

**Figure 2:**
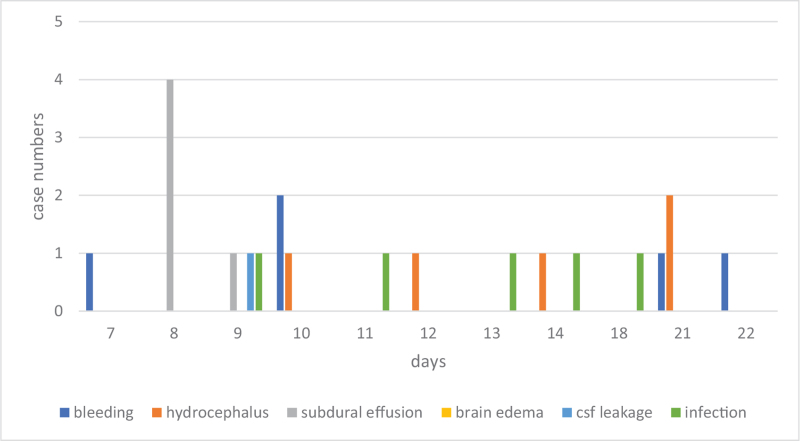
The distribution of the causes of return (cranial surgery) in late reoperation period (>7 days).

**Figure 3:**
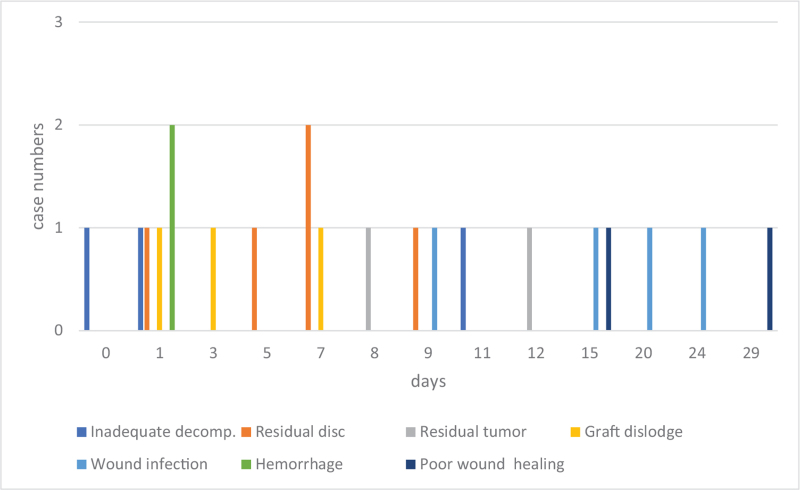
The distribution of the causes of return (spinal surgery) in the whole reoperation period.

**Figure 4:**
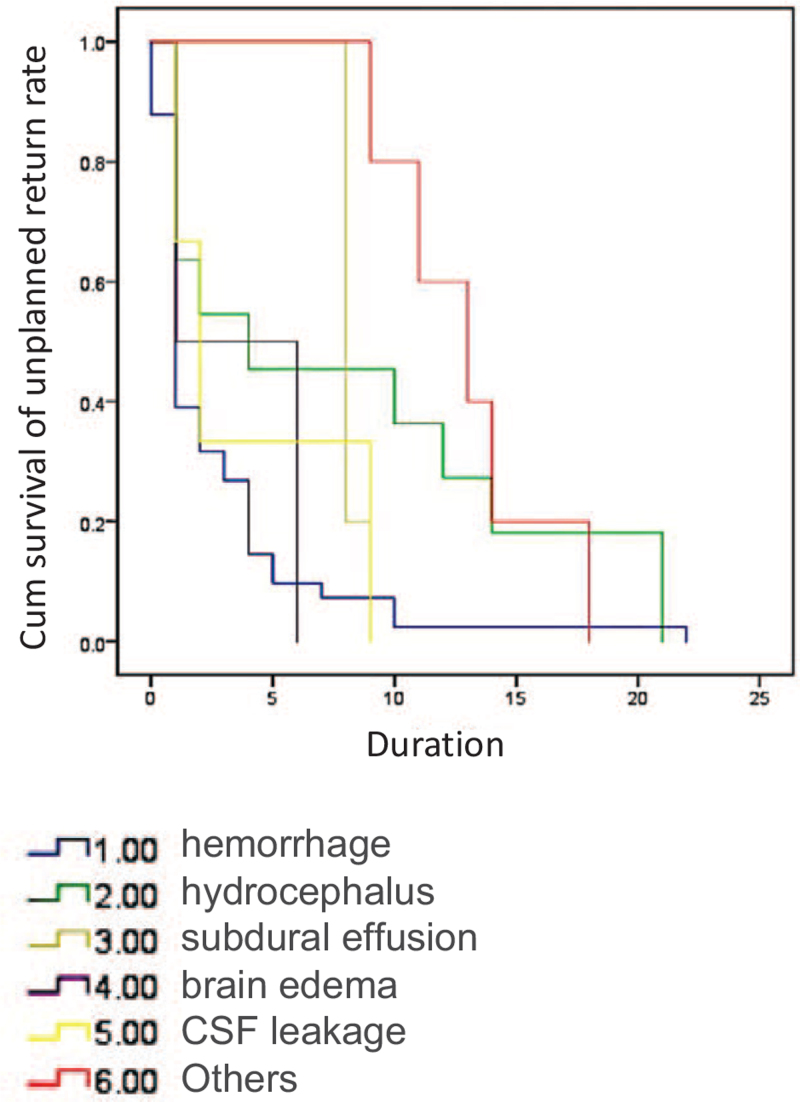
Time-event-analysis curve. Cox-regression model demonstrating days and the cause for reoperations.

As for cranial procedures, postoperative bleeding and hydrocephalus were the main reasons for early postoperative return. Eighty percent (37/46) of bleeding cases and 55% (6/11) of hydrocephalus cases were within postoperative day 6 (Fig. 1). On the other hand, 45% (5/11) of hydrocephalus, all subdural effusions (5/5), and all surgical site infections (5/5) were late postoperative returns (Fig. 2). As for spinal procedures, inadequate decompression (2), residual disc (2), graft dislodge (2), and hemorrhage (2) were the common reason for early postoperative return. Surgical site infection was the leading cause of late postoperative return (Fig. 3). However, there was no statistically significant difference between the different reasons for recovery and return period under the Cox regression test in time to event analysis (*P* = .1) (Fig. 4).

**Figure 5:**
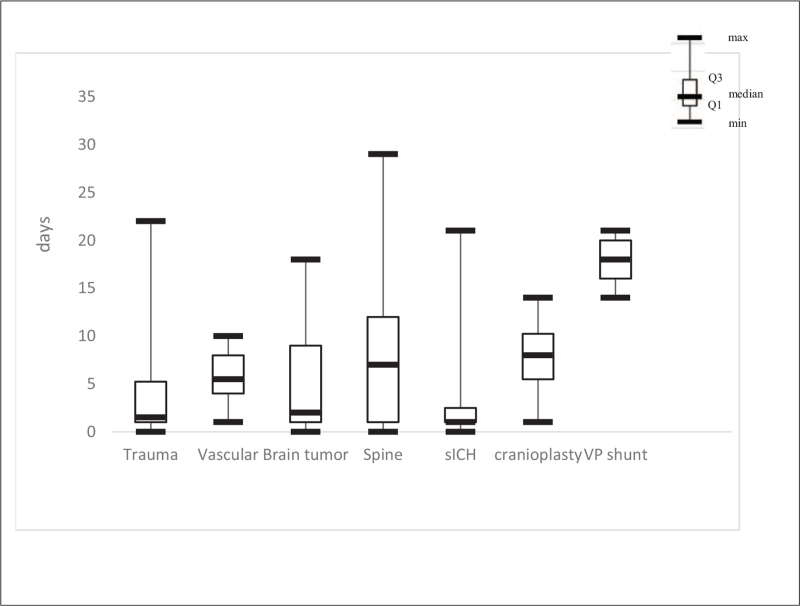
The Box plot showed the time interval distribution in each index surgery.

Traumatic brain injury surgery had a median time interval of 1.5 days, vascular surgery had a median time interval of 5.5 days, brain tumor surgery had a median time interval of 2 days, spine surgery had a median time interval of 7 days, spontaneous intracranial hemorrhage had a median time interval of 1 day, cranioplasty surgery had a median time interval of 8 days, and VP shunt surgery had a median time interval of 18 days (Fig. 5).

In this study, the incidence rate of early reoperation within 7 days was 1.26% (55/4365) in total and 59.1% (55/93) in reoperation patients within 30 days. Within 48 hours of the index surgery, the overall incidence rate of reoperation was 0.85% (37/4365) in total and 39.8% (37/93) in the reoperation group.

## 4 Discussion

The incidence of UROR has been proposed as a valuable indicator for assessing surgical quality.[^3^,^17^] However, there are four criteria that must be addressed for indicators: validity (better performance represents a better outcome), feasibility (it should be a common event, easily identifiable with administrative data), discriminability (the event is nondiscretionary, with no ambiguous situations in clinical practice, i.e., operating or not), and actionability (you can act according to this indicator, i.e., existing preventable factors).^[18]^

Three of these four criteria were fulfilled using the UROR rate as a quality indicator. However, actionability should be considered preventability. If the cause of the UROR could not be avoided, such as because of the disease's natural course, no action can be performed to improve this. For example, the leading cause of surgery-related returns in the above-mentioned cranial index surgeries is preventable hemorrhage. Brain retraction can be minimized by increasing cerebrospinal fluid drainage via external ventricular drainage for brain relaxation. This can, in turn, prevent postoperative hemorrhage over the surgical site.

The primary causes of surgery-related returns for spinal index surgery are poor graft dislodge, residual disc, and wound infection (Table 3). The surgeon can improve their skills by becoming more familiar with the instrument, sharing preoperative decision-making, and reinforcing the disinfection procedure.

### 4.1 Quality indicator for an internal audit

**Figure 6:**
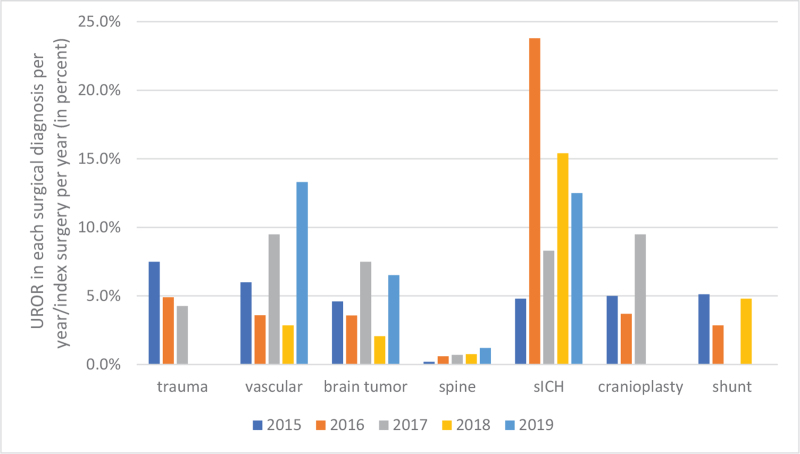
The trend in annual UROR rate in each surgical diagnosis.

“Unplanned return to the operating room rate” is a better surrogate than other indirect outcomes, such as readmission rate, length of hospital stay, and surgical site infection rate, as quality metrics.^[19]^ Comparing the data at the same hospital at different times can help minimize confounding factors, including the hospital's armamentarium, patient characteristics, and the surgeon's preference. In our study, the UROR rate decreased annually in traumatic brain injury and sICH but increased in vascular surgery, spine surgery, and cranioplasty. There was approximately the same UROR rate in brain tumors and VP shunt surgery (Fig. 6). According to the above trend, we can make an improvement from the modification of the intraoperative process or whether the decision-making from the different surgeons (threshold of the reoperation) was not the same.

### 4.2 Potential index surgery for external comparison

Comparing data between different institutions is difficult because of the age of the population, the disease's complexity and severity, and the follow-up period for each index surgery varies between hospitals.[^3^,^20^] Therefore, it is difficult to compare surgical quality between hospitals. For example, spontaneous ICH, vascular disease (AVM/aneurysm), or brain tumor with a lesion at different locations in the skull base or neurovascular involvement increases the complexity of the disease. Each hospital has a different complexity for the disease according to its ranking from referral. Determination of the unplanned return rate between hospitals or across the country requires case-mix correction, risk adjustment, and advanced statistical analyses for benchmarking. This requires systemic data collection and time-consuming analysis.^[21]^

Elective surgery with the need for implants, including instrumentation with cage, screw, and rod in spondylosis, CSF diversion with shunt, had fewer patient-related and disease-related factors. However, the UROR rate in spine surgery is not ideal for quality metrics because the rate is relatively low. Therefore, the statistical power was not sufficiently strong for comparison.

Shunt implantation surgery for CSF diversion is standard, easy to track, and suitable for comparison between hospitals.^[22]^ Almost all unplanned reoperation studies have mentioned comprehensive shunt implantation data. It has been proposed that the preventable shunt revision rate could be a quality metric used for external comparison in the pediatric population.[^23^,^24^,^25^] In the adult population, the preventable unplanned shunt implantation return rate has also been proposed as a possible external comparison quality metric.^[26]^ Reviewing the literature on UROR related to CSF diversion, Mukerji et al^[13]^ and Roy et al^[12]^ previously reported that shunt-related procedures accounted for 44% and 40.8% of all pediatric procedures at their study institutions, respectively. The rates of shunt-related unplanned reoperations among all unplanned reoperation patients were 78% and 73.5%, respectively. The overall rate of unplanned shunt-related reoperation among surgeries was 22% (91/410) in the 2-year follow-up in Mukerji et al's study,^[13]^ and 1.47% (117/7942) within 48 hours postoperatively in Roy et al's study.^[12]^ McLaughlin et al reviewed their shunt failure cases, which accounted for 34.4% (63 [pedi/adult: 36/27]/183) of all early unplanned returns and 0.39% (27/6912) among all surgeries within 7 days post-operation.^[14]^ Panagiotis et al reported that the rate of shunt-related unplanned reoperation among all unplanned reoperation patients was 13.5% (42/311) and 0.46% (42/9200) among all surgeries within 45 days.^[27]^ A comparison of our study with other studies using shunt surgery data is shown in Table 4.

**Table 4:** Characteristics of studies over shunt reoperation.

	Years of publication	Type of study	Shunt procedures/total cases (%)	Shunt reop/total cases	Shunt reop/overall unplanned surgery	Population	f/u duration
Mukerji et al	2012	retrospective	44	22%(91/410)	78%	Pediatrics	2 years
Roy et al	2017	retrospective	40.8	1.47%(117/7942)	73.5%	Pediatrics	48 h
Mc Laughlin et al	2015	retrospective	Nil	0.39%(27/6912) for adult	34.4%(63/183)	(pedi/adult- 36:27)	7 days
Panagiotis et al	2018	retrospective	4.3	0.46%(42/9200)	13.5%(42/311)	Adult	45 days
Eric et al	2020	retrospective	4.2	0.37%(14/3760)	4.1%(14/342)	Adult	30 days
Our study		retrospective	3.06	0.017%(1/6309)	0.69%(1/145)	Adult	30 days

### 4.3 Data processing and real-time review for feedback

There is an issue with the computer-determined true or false UROR rate due to misidentified coding in the electronic medical record system and inadequate documentation by the surgeon in the notes. Yihan et al reported that only 64.7% was true UROR from NSQIP-identified UROR, and 60% of the reason for the “false UROR rate” was inadequate documentation.^[16]^ The correct assignment requires a time-consuming manual, secondary chart review for validation, and the data cannot be presented in real time. The development of a real-time business intelligence analysis system can decrease documentation and recall errors. Panagiotis et al presented their data using real-time surgical data rather than retrospective analysis; their system could also automatically send confirmation emails to the surgeon to confirm whether the surgery was planned before the final entry into the database.^[27]^ Quick acquisition and analysis of the data is a requirement for meaningful feedback to the team or individual, especially within the context of business intelligence for healthcare delivery. Decision makers need precise information and streamlined charts to determine how to deliver safer and more cost-effective care.[^28^,^29^]

### 4.4 Limitations

The present study has several limitations. First, the studied complication numbers were rather small, but the overall number of neurosurgical operations was large. Thus, there is insufficient statistical power to demonstrate a significant difference. Second, this was a retrospective case series. The validity of preventability determination requires a root-cause analysis prospectively. Third, instead of data mining from the electronic medical records via coding or a built-in UROR reporting function, the data were collected from weekly EXCEL files from previous chart rounds, and minor surgeries, such as changing EVD or ICP monitoring procedures, were not included in the analysis.

## 5 Conclusion

In summary, we analyzed 4365 procedures in five consecutive years, and an overall UROR rate of 2% was reported within 30 days of the index operation. Hemorrhage (46/93, 49.5%) was the leading cause of all UROR, accounting for 80% (37/46) of occurrences within 7 days post-operation. Regular tracking of the UROR rate for each index procedure and identifying preventable causes, as determined by comprehensive analysis, can help doctors and hospitals make sustainable improvements.

## Author contributions

**Conceptualization:** weichao Huang, Martin Hsiu-Chu Lin.

**Data curation:** weichao Huang, Yin-Ju Chen.

**Formal analysis:** weichao Huang.

**Investigation:** Yin-Ju Chen.

**Methodology:** weichao Huang, Martin Hsiu-Chu Lin.

**Software:** weichao Huang, Martin Hsiu-Chu Lin.

**Supervision:** Martin Hsiu-Chu Lin, Ming-Hsueh Lee.

**Writing – original draft:** weichao Huang.

**Writing – review & editing:** weichao Huang.
